# Insertion Torque Characteristics of the KS 3 Implant in Weak Bone, Standardized Extraction-Socket-like, and Maxillary Sinus Simulation Models: An In Vitro Comparative Study

**DOI:** 10.3390/bioengineering13060705

**Published:** 2026-06-19

**Authors:** Na Ri Seo, Ye-Seul Jung, Dayeon Park, Jisung Kim, Dong-Wook Han, Bongju Kim

**Affiliations:** 1 Program in Neuroscience, Department of Dental Science, Graduate School, Seoul National University, Seoul 03080, Republic of Korea; tj0943@snu.ac.kr; 2 Dental Life Science Research Institute, Seoul National University Dental Hospital, Seoul 03080, Republic of Korea; jyseul@snu.ac.kr (Y.-S.J.); dayeonp740@gmail.com (D.P.); 3 Department of Oral and Maxillofacial Surgery, School of Dentistry, Seoul National University, Seoul 03080, Republic of Korea; jisung920415@snu.ac.kr; 4 Department of Cogno-Mechatronics Engineering, Pusan National University, Busan 46241, Republic of Korea; 5 School of Transdisciplinary Engineering, University College, Pusan National University, Busan 46241, Republic of Korea

**Keywords:** dental implant, insertion torque, insertion resistance, weak bone, extraction-socket-like model, maxillary sinus, implant macrodesign, in vitro study

## Abstract

Objective: This in vitro study evaluated the insertion torque characteristics of the KS 3 implant compared with the TSIII implant in standardized artificial bone models representing weak bone, extraction-socket-like reduced support, and maxillary sinus simulation conditions. Materials and Methods: A comparative in vitro study was performed using three models: a weak bone model, a standardized extraction-socket-like reduced-support model, and a maxillary sinus simulation model. Maximum and final insertion torque values were obtained from torque–depth curves. Torque–depth integrals were additionally calculated as exploratory secondary parameters. Statistical analyses were performed using Welch’s *t*-test and two-way ANOVA where appropriate, and the results were interpreted as exploratory because of the limited sample size. Results: The KS 3 implant showed higher maximum and/or final insertion torque values than the TSIII implant in the weak bone, extraction-socket-like, and maxillary sinus simulation models. In the maxillary sinus model, the torque values showed directional differences according to implant type and residual bone height under the tested fixed undersized drilling protocols for both CAS drilling and bone compaction drilling. Torque–depth integral analysis provided additional information regarding cumulative insertion resistance. Conclusions: Within the limitations of this controlled in vitro study, the KS 3 implant showed higher insertion torque values than the TSIII implant under the tested artificial bone conditions. These findings should be interpreted as in vitro insertion torque data under the tested artificial bone and drilling conditions, not as evidence of clinical superiority. In the maxillary sinus simulation model, the observed torque differences should be interpreted as the combined effect of implant macrodesign and the fixed undersized drilling protocol, rather than as an isolated macrodesign effect.

## 1. Introduction

Dental implants have been widely accepted as a predictable and effective treatment option for the rehabilitation of partially and completely edentulous patients. Nevertheless, implant placement in mechanically compromised conditions, such as weak bone, fresh extraction sockets, and posterior maxillary sites with limited residual bone height, continues to present a substantial biomechanical challenge. In these situations, the available circumferential bone support is reduced, the stiffness of the surrounding substrate is lower, and resistance to implant advancement is diminished. As a result, the capacity of the implant to establish adequate primary stability becomes a critical determinant of treatment predictability [1,2,3,4,5]. In the present study, this clinical scenario was not reproduced as a true anatomical extraction socket, but was approximated using a standardized extraction-socket-like reduced-support model.

From a biomechanical perspective, primary stability may be understood as the immediate mechanical restraint generated by frictional resistance, compressive interlocking, and thread-mediated engagement during implant insertion. This restraint determines the degree to which displacement and micromotion can be limited before biologic fixation is established. Insertion torque is therefore relevant not merely as an operative parameter, but as a surrogate of rotational resistance and interface engagement during implant placement. At the same time, insertion torque should not be interpreted as a complete synonym for stability, because its relationship with stability is modulated by bone quality, implant macrodesign, and the measurement method used [3,4,5,6,7,8,9,10].

Previous studies have shown that implant geometry can significantly alter the mechanical behavior achieved during insertion. Tapered bodies, progressive thread profiles, condensing designs, and macrogeometry intended to increase apical or coronal interference have all been proposed as strategies to improve primary mechanical anchorage, particularly in low-density bone [6,7,8,11,12,13,14,15,16,17,18]. In addition, insertion-related stability is influenced by osteotomy preparation, as drilling undersizing, compaction protocols, and residual bone support may alter torque generation and bone deformation at the implant–bone interface [9,16,17].

These biomechanical issues become even more relevant in extraction socket and maxillary sinus-related indications. In immediate-placement situations, implant stability must often be obtained from the apical and peripheral socket walls rather than from uniform circumferential bone support. Likewise, in sinus-related placement, reduced residual bone height limits the effective engagement length and diminishes the structural support available for initial fixation. Previous ex vivo and clinical studies have shown that both implant macrodesign and residual bone height substantially influence primary stability in these scenarios [15,17,19,20].

A previous study evaluated screw-joint behavior in the same implant platform and reported differences in abutment screw torque loss under cyclic fatigue loading [21]. However, screw-joint behavior and fixture-level insertion torque represent different mechanical outcomes. Therefore, the present study was designed as an independent in vitro evaluation of fixture-level insertion torque behavior under standardized artificial bone and drilling conditions.

The KS 3 implant platform has macrodesign features intended to influence insertion behavior in weak bone, extraction-socket-like, and maxillary sinus simulation conditions. Previous studies have reported that tapered implant bodies and thread-related macrodesign features can affect insertion torque and primary mechanical stability, particularly in low-density bone [6,7,8,18]. In sinus-related models, residual bone height and implant macrodesign have also been shown to influence primary stability [15,17]. In addition, drilling undersizing and compaction protocols can modify torque generation and bone deformation at the implant–bone interface [9,16,22,23]. Therefore, macrodesign changes that affect thread engagement, taper-mediated compression, and coronal or apical load transfer may alter the torque–depth behavior recorded during implant insertion.

In the present study, the term “macrodesign” refers specifically to measurable external fixture design parameters, including implant length and diameter, maximum thread diameter, thread pitch, upper and lower thread depth, body/core taper, apical geometry, and surface configuration. The KS 3 implant was compared with the TSIII implant using implants of the same nominal diameter and length, while differences in thread pitch, lower thread depth, and core taper were considered as the primary macrodesign modifications.

Therefore, the aim of the present study was to evaluate the insertion characteristics of the KS 3 implant in weak bone, standardized extraction-socket-like reduced-support, and maxillary sinus simulation models.

## 2. Materials and Methods

### 2.1. Study Design

A comparative in vitro study was performed using three insertion models: a weak bone model, a standardized extraction-socket-like reduced-support model, and a maxillary sinus model. Maximum insertion torque and final insertion torque were used as the primary outcome variables. For each model condition, independent implants were used, and each implant was tested only once. Four independent implants were evaluated for each implant type under each experimental condition, except for the weak bone model, in which five independent implants were evaluated for each implant type. Torque–depth curves were recorded during implant placement, and the resulting values were used for statistical comparison between groups.

### 2.2. Implant Systems

**Figure 1:**
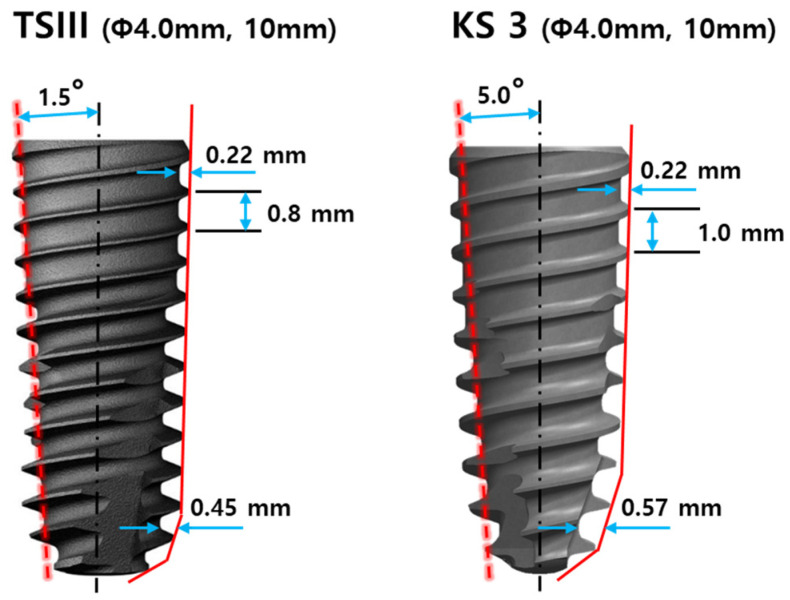
Macrodesign characteristics of the tested implant systems. The TSIII and KS 3 implants had the same nominal dimension (Ø4.0 × 10 mm) and SA surface treatment. The schematic illustrates the major external macrodesign parameters evaluated in this study, including body/core taper, thread pitch, upper thread depth, and lower thread depth. The TSIII implant had a 1.5° core taper, 0.8 mm thread pitch, 0.22 mm upper thread depth, and 0.45 mm lower thread depth, whereas the KS 3 implant had a 5.0° core taper, 1.0 mm thread pitch, 0.22 mm upper thread depth, and 0.57 mm lower thread depth.

The tested implants were TSIII SA Implant and KS 3 SA Implant (Osstem Implant Co., Ltd., Seoul, Republic of Korea; catalogue No. TS3S4010S for TSIII, KS3S4010S for KS 3). Both implants had the same nominal diameter and length (Ø4.0 × 10 mm) and the same SA surface treatment. The evaluated macrodesign differences included thread pitch, lower thread depth, and body/core taper. The maximum outer diameter was Ø4.25 mm for both systems. The thread pitch was 0.8 mm in the TSIII implant and 1.0 mm in the KS 3 implant. The lower thread depth was 0.45 mm in the TSIII implant and 0.57 mm in the KS 3 implant. The core taper was 1.5° in the TSIII implant and 5.0° in the KS 3 implant (Figure 1). The implants and drills were provided by Osstem Implant Co., Ltd., for research purposes; the manufacturer had no role in the study design, data acquisition, data analysis, data interpretation, or manuscript preparation.

**Figure 2:**
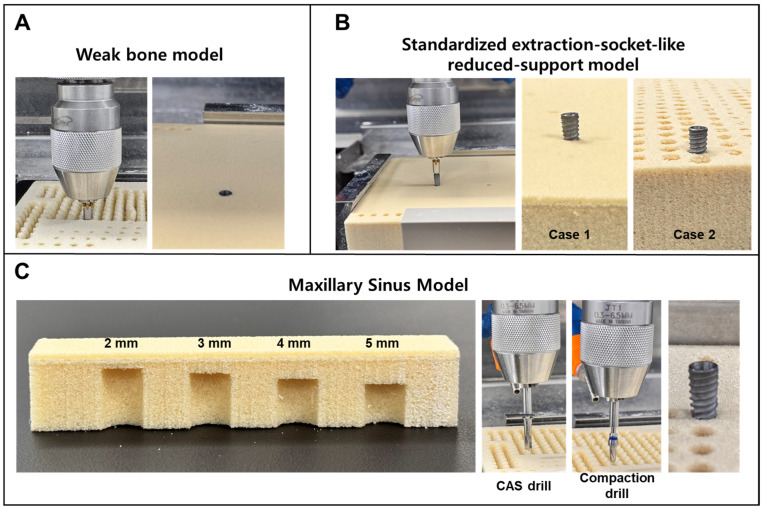
Photographs of the artificial bone models and implant insertion procedures. (**A**) Weak bone model showing implant insertion and the prepared osteotomy. (**B**) Standardized extraction-socket-like reduced-support model showing implant insertion and the two artificial bone conditions. Case 1 consisted of a 1.0 mm #20 cortical layer over #10 cancellous polyurethane foam, whereas Case 2 consisted entirely of #20 cancellous polyurethane foam. (**C**) Maxillary sinus simulation model prepared with residual bone heights of 2, 3, 4, and 5 mm. The CAS drilling procedure, bone compaction drilling procedure, and implant placement after osteotomy preparation are also shown.

Representative photographs of the artificial bone models and implant insertion procedures are shown in Figure 2.

### 2.3. Weak Bone Model

The weak bone model was prepared using a rigid polyurethane foam block conforming to ASTM F1839 (Sawbones, Pacific Research Laboratories, Vashon, WA, USA; catalogue No. 1522-105, 1522-751, 1522-1199). The model consisted of a 1.0 mm cortical layer with a density grade of #20 (20 lb/ft^3^; 0.32 g/cm^3^) over cancellous foam with a density grade of #10 (10 lb/ft^3^; 0.16 g/cm^3^). This configuration was selected to simulate a low-density bone condition. Osteotomies were prepared according to the manufacturer’s drilling protocol using the Ø2.0/2.5 × 10 side-cut drill (Osstem Implant Co., Ltd., Seoul, Republic of Korea; catalogue No. OSLMD20L) followed by an F3.5 × 10 122 Taper 3510 drill (Osstem Implant Co., Ltd., Seoul, Republic of Korea; catalogue No. 122TPD3510). The common drilling device, drilling speed, torque-monitoring condition, and irrigation procedure are described in Section 2.7. The final drilling depth was 10 mm, and implants were inserted to a depth of 10 mm at the bone level, with an additional 1 mm subcrestal insertion to minimize the influence of the cortical bone layer on the measured insertion torque. For each implant type, five independent implants were inserted under the weak bone condition, and each implant was tested only once. The resulting torque–depth curves were used to derive the maximum insertion torque and final insertion torque.

### 2.4. Standardized Extraction-Socket-like Reduced-Support Model

A standardized extraction-socket-like reduced-support model was prepared to reproduce a limited insertion-depth condition rather than the full anatomical morphology of a clinical extraction socket. Two artificial bone conditions were used: Case 1 consisted of a 1.0 mm #20 cortical layer over #10 cancellous foam, and Case 2 consisted entirely of #20 cancellous foam. The term “extraction-socket-like” was used because the model simulated reduced coronal support and limited insertion depth, but did not reproduce socket wall convergence, buccal plate defects, or patient-specific socket morphology. Drilling was performed using an F2.2 twist drill to a depth of 5.66 mm, which allowed a final insertion depth of 5 mm, under the common drilling device, drilling speed, torque-monitoring condition, and irrigation procedure described in Section 2.7. For each implant type and each extraction-socket-like condition, four independent implants were tested, and each implant was inserted only once. Maximum insertion torque and final insertion torque were derived from the recorded torque–depth curves.

### 2.5. Maxillary Sinus Model

The maxillary sinus simulation model was prepared using a soft artificial bone block consisting of a 1.0 mm #20 cortical layer and #10 cancellous foam. To avoid deformation of thin residual bone during drilling, osteotomy preparation was first performed in a block thicker than 10 mm. After drilling, the cancellous portion beneath the osteotomy was trimmed to create residual bone heights of 2, 3, 4, and 5 mm. This procedure allowed standardized osteotomy preparation while simulating limited residual bone support beneath the cortical layer.

Two drilling protocols were evaluated as separate experimental conditions, not as sequential procedures. In the CAS drilling condition, the final osteotomy was prepared using a Ø2.8 CAS drill (Osstem Implant Co., Ltd., Seoul, Republic of Korea; catalogue No. OSNDR2813TL). In the bone compaction condition, the osteotomy was prepared using an F4.0 × 5 compaction drill (Osstem Implant Co., Ltd., Seoul, Republic of Korea; catalogue No. BCD4005) according to the manufacturer’s protocol. Both CAS drilling and bone compaction drilling were performed under the common drilling device, drilling speed, torque-monitoring condition, and irrigation procedure described in Section 2.7. Because accurate hole formation was difficult in the initially prepared thin-bone model due to bending of the artificial bone during drilling, the protocol was refined so that drilling was first performed in bone thicker than 10 mm, after which the remaining bone thickness was adjusted to the designated residual height before implant insertion. For each implant type and each residual bone-height condition, four independent implants were tested, and each implant was inserted only once. Maximum and final insertion torque values were extracted from the recorded torque–depth curves, and final insertion torque was used as the primary comparative outcome for factorial analysis. Because the sinus model used fixed undersized osteotomy protocols, this model was designed to evaluate insertion torque behavior under the tested manufacturer-specified drilling conditions rather than to isolate the independent effect of implant macrodesign alone.

### 2.6. Outcome Variables

The primary outcome variables were maximum insertion torque and final insertion torque, both expressed in Ncm. In the weak bone and standardized extraction-socket-like reduced-support models, these values were extracted from torque–depth curves generated during implant insertion. In the maxillary sinus model, both maximum and final insertion torque values were extracted from the torque–depth curves. Final insertion torque was used as the primary comparative outcome for factorial analysis because the main purpose of this model was to compare insertion resistance at the final seating position under different residual bone heights and drilling protocols. In addition to maximum and final insertion torque, the torque–depth integral was calculated from the recorded torque–depth curves as an exploratory secondary parameter. The integral was obtained by numerically integrating torque values over insertion depth using the trapezoidal rule and was expressed as Ncm·mm. The depth–torque recordings were exported at the original acquisition resolution without smoothing or averaging. The depth interval used for numerical integration was determined from the recorded depth values in the raw data file, and the trapezoidal rule was applied to consecutive recorded depth–torque points. Integration was performed from 0 mm to the final recorded insertion depth for each implant insertion. Therefore, the integration range differed by model: approximately 5 mm in the standardized extraction-socket-like reduced-support model and approximately 10 mm in the weak bone and maxillary sinus simulation models. For this reason, torque–depth integral values were interpreted only within the same model condition and were not directly compared across different model categories. The raw data file includes insertion depth and torque values for each independent implant insertion. The exported depth values were used directly for numerical integration. Because the first recorded depth point did not always begin at 0 mm, a zero-torque value at 0 mm was added before numerical integration. This parameter was used as a surrogate estimate of cumulative insertion resistance during implant placement. The replicate-level raw torque–depth data used to calculate maximum insertion torque, final insertion torque, and torque–depth integrals are provided as a machine-readable Supplementary Data Files (CSV format). This file includes the depth- and torque-recording data for each independent implant insertion, allowing verification of the values reported in the main tables and Supplementary Table S1.

### 2.7. Drilling, Implant Insertion, and Torque Measurement System

Osteotomy preparation and implant insertion were performed using the same customized automated implant insertion testing device (GS Co., Yongin, Republic of Korea), which was equipped with a motor-driven drilling/insertion unit to maintain a constant drilling and insertion axis. Osteotomy drilling was performed under continuous irrigation at 1200 rpm. A fixed drilling torque was not preset as an experimental target, and drilling torque was not analyzed as an outcome variable in this study. During drilling and implant insertion, torque was monitored using the same calibrated torque measurement system equipped with a Kistler 4502A torque sensor (Kistler, Winterthur, Switzerland). The torque sensor used in this study had a maximum measurement range of 200 Ncm and a specified measurement accuracy/resolution of ±0.2% of full scale, corresponding to approximately ±0.4 Ncm. The sensor provided an analog output signal of ±5 V at the rated torque and was calibrated before testing according to the manufacturer’s specifications.

After osteotomy preparation, the osteotomy site was kept moist until implant insertion. Implant insertion was performed using the same customized automated device at 15 rpm, and the axial insertion load was maintained at approximately 10 N throughout insertion. All experiments were performed at room temperature under controlled laboratory conditions (25 °C, 40–60% relative humidity). Torque signals were recorded continuously and converted into torque–depth curves in real time. Maximum insertion torque and final insertion torque were extracted from these recorded curves for further analysis. All measurements were performed under consistent drilling and insertion conditions to minimize procedural variability. All drilling and insertion procedures were performed by a single trained operator using the same automated device and torque-measurement setup. The testing order of implant type and experimental condition was randomized before insertion using a computer-generated random sequence to reduce order-related bias. Blinding to implant identity was not feasible because the implant macrodesigns were visually distinguishable.

To address concerns regarding the metrological basis of the torque measurements and the low within-condition variability observed in the maxillary sinus model, the measurement chain, exported depth–torque recordings, and raw data extraction process were reviewed. Replicate-level raw data were verified against the exported unprocessed depth–torque recordings, confirming that all reported values were directly traceable to the machine-readable Supplementary Data Files. No smoothing, averaging, filtering, or derived summary curves were used to generate the tabulated maximum and final insertion torque values. The same customized automated device, torque sensor, acquisition settings, calibration procedure, drilling/insertion axis, irrigation condition, and trained operator were used across all experimental conditions. Osteotomy preparation was performed using the same mechanically guided setup and the same drilling protocols to minimize geometric variation in drilling axis and depth.

The polyurethane foam blocks used in this study were purchased for the present experimental series using the catalogue numbers listed in Supplementary Tables S2–S4. However, purchase documents and lot identifiers were not retained in the experimental records; therefore, the lot numbers of the Sawbones #10 and #20 polyurethane foam blocks could not be independently verified. This limitation was considered when interpreting the unusually low within-condition variability observed in some maxillary sinus model cells.

In several sinus-model conditions, the numerical dispersion of terminal final-torque values was smaller than the specified accuracy of the torque sensor. These values were therefore not interpreted as evidence of metrological precision beyond the declared sensor accuracy. Instead, they were treated only as numerical dispersion of exported terminal readings obtained under a highly constrained machine-guided acquisition setup. Accordingly, the low variability observed in some sinus-model conditions should be interpreted within this automated laboratory setup, and the corresponding statistical outputs should be regarded as setup-specific exploratory descriptors rather than generalized to clinical bone conditions or less controlled experimental settings.

### 2.8. Statistical Analysis

All data were analyzed using SigmaPlot 14 (Systat Software Inc., San Jose, CA, USA). Descriptive statistics are presented as mean ± standard deviation. Because of the limited sample size and controlled in vitro setting, the statistical analyses were interpreted as exploratory rather than confirmatory. Because unusually low within-condition variability was observed in some maxillary sinus model conditions, the corresponding inferential statistics were interpreted with particular caution. The statistical results were used to describe the direction and consistency of the observed torque differences under the tested setup, rather than to support generalizable confirmatory claims. Normality of data distribution was assessed using the Shapiro–Wilk test. However, the results were interpreted cautiously because of the small sample size. For the weak bone model, between-implant comparisons were performed using Welch’s *t*-test. For the standardized extraction-socket-like reduced-support model, Welch’s *t*-test was used with Holm–Bonferroni adjustment for multiple pairwise comparisons. For the maxillary sinus model, the effects of implant type and residual bone height on final insertion torque were evaluated separately for CAS drilling and bone compaction drilling using two-way ANOVA. For the maxillary sinus model, pairwise between-implant comparisons at each residual bone height were treated as descriptive. To avoid overinterpretation of multiple unadjusted pairwise tests, pairwise *p*-values were not tabulated in Tables 3 and 4. The tables instead report cell means, standard deviations, mean differences, and 95% confidence intervals. The primary model-level analysis was based on two-way ANOVA and was interpreted cautiously because of the limited sample size and the low within-condition variability observed in some cells. Because the unusually low within-condition variability in several sinus-model cells may inflate inferential statistics, the ANOVA outputs were not interpreted as definitive estimates of population-level effects. For the torque–depth integral, between-implant comparisons were performed within each experimental condition using Welch’s *t*-test and were considered exploratory. Where *p*-values are reported in the manuscript or Supplementary Table S1, they are presented as descriptive statistical outputs rather than as confirmatory evidence. All summary statistics were calculated from the replicate-level raw torque–depth data provided in the machine-readable Supplementary File.

## 3. Results

### 3.1. Weak Bone Model

**Figure 3:**
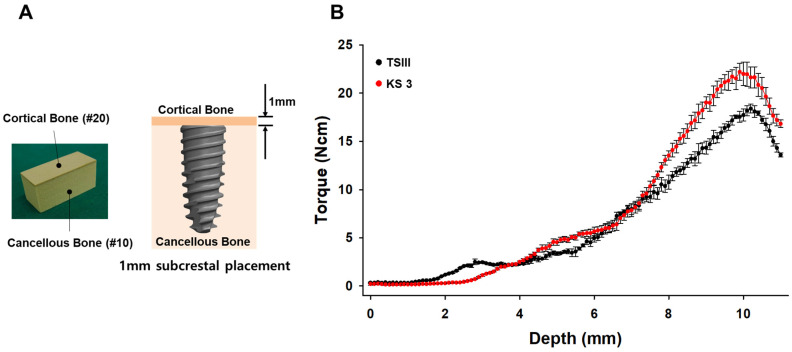
Weak bone insertion model and torque–depth curves. (**A**) Schematic illustration of the weak bone model. The model consisted of a 1.0 mm #20 cortical layer over #10 cancellous polyurethane foam. Osteotomy preparation was performed using a side-cut drill followed by a 122 Taper 3510 drill. Implants were inserted to a depth of 10 mm at the bone level, with an additional 1 mm subcrestal placement to reduce the influence of the cortical bone layer on insertion torque. (**B**) Torque–depth curves of the TSIII and KS 3 implants in the weak bone model.

In the weak bone model, higher insertion-related torque values were observed for the KS 3 implant than for the TSIII implant (Table 1, Figure 3). The maximum insertion torque increased from 18.61 ± 0.36 Ncm in the TSIII implant to 22.51 ± 0.93 Ncm in the KS 3 implant (mean difference: 3.90 Ncm; 95% CI: 2.77–5.03; *p* = 0.000260). Likewise, the final insertion torque increased from 13.59 ± 0.22 Ncm to 16.82 ± 0.39 Ncm (mean difference: 3.23 Ncm; 95% CI: 2.74–3.72; *p* = 0.00000250).

### 3.2. Standardized Extraction-Socket-like Reduced-Support Model

**Figure 4:**
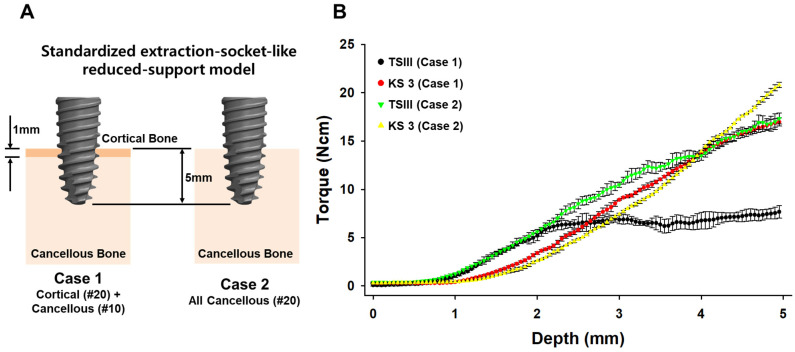
Standardized extraction-socket-like reduced-support model and torque–depth curves. (**A**) Schematic illustration of the two artificial bone conditions used in this model. Case 1 consisted of a 1.0 mm #20 cortical layer over #10 cancellous polyurethane foam, whereas Case 2 consisted entirely of #20 cancellous polyurethane foam. This model was designed to reproduce a standardized reduced-support and limited insertion-depth condition rather than the full anatomical morphology of a clinical extraction socket. (**B**) Torque–depth curves of the TSIII and KS 3 implants at a 5 mm insertion depth. Osteotomy preparation was performed using an F2.2 twist drill to a depth of 5.66 mm.

In the standardized extraction-socket-like reduced-support model, the KS 3 implant demonstrated higher insertion-related torque values than the TSIII implant in both tested conditions (Table 2, Figure 4). In Case 1, insertion torque increased from 7.89 ± 0.68 Ncm in the TSIII implant to 17.13 ± 0.44 Ncm in the KS 3 implant, with a mean difference of 9.25 Ncm (95% CI: 8.22–10.27; Holm-adjusted Welch’s *t*-test, *p* = 0.000004). In Case 2, insertion torque increased from 17.60 ± 0.46 Ncm to 21.32 ± 0.26 Ncm, with a mean difference of 3.72 Ncm (95% CI: 3.02–4.41; Holm-adjusted Welch’s *t*-test, *p* = 0.000050). Because the implants were inserted only to a limited depth of 5 mm in this model, the peak torque occurred at the final insertion depth; therefore, the maximum and final insertion torque values were operationally identical in all conditions.

### 3.3. Maxillary Sinus Model: CAS Drilling

**Figure 5:**
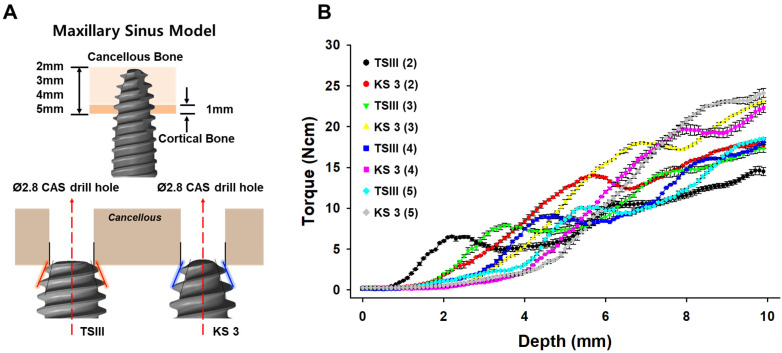
Maxillary sinus simulation model with CAS drilling and torque–depth curves. (**A**) Schematic illustration of the maxillary sinus simulation model. The model consisted of a 1.0 mm #20 cortical layer and #10 cancellous polyurethane foam, with residual bone heights of 2, 3, 4, and 5 mm. Osteotomy preparation was performed using a Ø2.8 CAS drill, and the same final osteotomy diameter was applied to both the TSIII and KS 3 implant groups. (**B**) Torque–depth curves of the TSIII and KS 3 implants under CAS drilling at each residual bone height.

Under CAS drilling, directionally higher maximum and final insertion torque values were observed for the KS 3 implant than for the TSIII implant at all tested residual bone heights (Table 3, Figure 5). The two-way ANOVA was used as the primary model-level analysis for final insertion torque, but the results were interpreted cautiously because of the limited sample size and the low within-condition variability observed in some cells. Directional effects of implant type, residual bone height, and their interaction were observed under the tested setup. Pairwise between-implant comparisons at individual residual bone heights were treated as descriptive. Overall, under the tested fixed undersized CAS drilling protocol, the KS 3 implant showed higher maximum and final insertion torque values than the TSIII implant across all tested residual bone heights.

### 3.4. Maxillary Sinus Model: Bone Compaction Drilling

**Figure 6:**
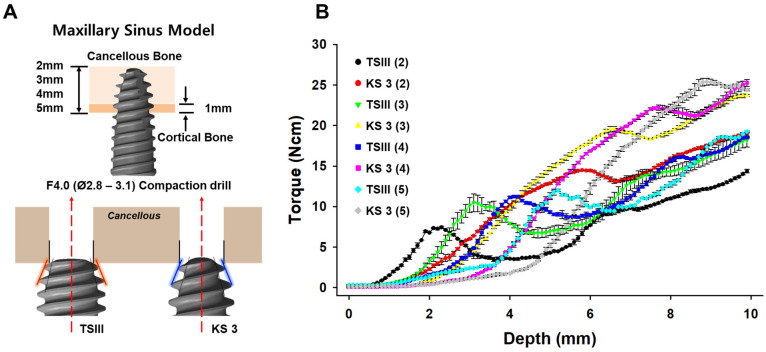
Maxillary sinus simulation model with bone compaction drilling and torque–depth curves. (**A**) Schematic illustration of the bone compaction drilling condition. The maxillary sinus simulation model consisted of a 1.0 mm #20 cortical layer and #10 cancellous polyurethane foam, with residual bone heights of 2, 3, 4, and 5 mm. Bone compaction drilling was evaluated as a separate experimental protocol from CAS drilling, and the final osteotomy was prepared using an F4.0 (Ø2.8–3.1) × 5 compaction drill according to the manufacturer’s protocol. (**B**) Torque–depth curves of the TSIII and KS 3 implants under bone compaction drilling at each residual bone height.

With bone compaction drilling, directionally higher maximum and final insertion torque values were observed for the KS 3 implant than for the TSIII implant at all tested residual bone heights (Table 4, Figure 6). The two-way ANOVA was used as the primary model-level analysis for final insertion torque, but the results were interpreted cautiously because of the limited sample size and the low within-condition variability observed in some cells. Directional effects of implant type, residual bone height, and their interaction were observed under the tested setup. Pairwise between-implant comparisons at individual residual bone heights were treated as descriptive. Overall, under the tested fixed undersized bone compaction drilling protocol, the KS 3 implant showed higher maximum and final insertion torque values than the TSIII implant across all tested residual bone heights.

### 3.5. Overall Trend Across Models

When the results from all models were considered together, the KS 3 implant showed a consistent tendency toward higher insertion-related torque values than the TSIII implant. In the weak bone and standardized extraction-socket-like reduced-support models, this tendency was reflected in both maximum insertion torque and final insertion torque (Table 1 and Table 2; Figure 3 and Figure 4). In the maxillary sinus model, the same directional trend was observed in final insertion torque under both tested fixed undersized drilling protocols (Table 3 and Table 4; Figure 5 and Figure 6). Overall, the two implant systems showed different insertion torque responses under the tested reduced-support artificial bone and fixed drilling conditions, with higher maximum and/or final insertion torque values observed for the KS 3 implant in the tested models. Torque–depth integrals were additionally calculated from the recorded torque–depth curves as exploratory secondary parameters (Supplementary Table S1). Because the experimental models differed in insertion depth, drilling protocol, and residual bone height, the torque–depth integral values were interpreted within each model condition rather than compared directly across different models. In most tested conditions, the KS 3 implant showed a higher torque–depth integral than the TSIII implant. However, in Case 2 of the standardized extraction-socket-like reduced-support model, the TSIII implant showed a higher torque–depth integral despite the higher final insertion torque observed for the KS 3 implant. This finding indicates a dissociation between cumulative insertion resistance and final seating torque in this condition.

## 4. Discussion

The present study was not designed to establish clinical superiority of the KS 3 implant. Rather, it was intended as a controlled in vitro comparison of insertion torque behavior under standardized artificial bone conditions. Therefore, the findings should be interpreted as in vitro insertion torque data obtained under the tested artificial bone and drilling conditions, not as direct evidence of improved osseointegration, implant survival, or clinical success. The present study was conducted to compare insertion torque behavior between the KS 3 and TSIII implants under mechanically compromised artificial bone conditions. The principal finding was that the KS 3 implant demonstrated higher insertion-related torque values than the TSIII implant in the weak bone, standardized extraction-socket-like reduced-support, and maxillary sinus simulation models. In the weak bone and standardized extraction-socket-like reduced-support models, this tendency was reflected in both maximum insertion torque and final insertion torque, whereas in the maxillary sinus model, consistently higher final insertion torque values were observed under both CAS drilling and bone compaction drilling (Table 1, Table 2, Table 3 and Table 4; Figure 3, Figure 4, Figure 5 and Figure 6). These findings indicate that insertion resistance differed between the two implant systems under the tested artificial bone and drilling conditions. However, particularly in the maxillary sinus simulation model, the observed torque differences should be interpreted as the combined effect of implant geometry, residual bone height, and the selected undersized drilling protocol, rather than as an isolated macrodesign effect. This interpretation is consistent with previous studies showing that implant geometry, thread configuration, bone density, osteotomy preparation, and drilling undersizing can influence insertion torque and primary mechanical engagement during implant placement [6,7,8,9,11,12,13,14,15,16,17,18,22,23].

The present study should be interpreted independently as an in vitro evaluation of fixture-level insertion torque behavior under standardized artificial bone and drilling conditions. Although previous mechanical studies have evaluated other aspects of implant system performance, the present outcomes are limited to insertion torque and torque–depth behavior at the fixture–substrate interface. Therefore, the findings should not be interpreted as part of a product-level validation sequence, but as exploratory mechanical data generated under the specific experimental conditions used in this study.

From a biomechanical perspective, insertion torque is influenced by the interaction among implant macrogeometry, substrate stiffness, and osteotomy preparation. When bone density is low or the available supporting bone volume is limited, thread-mediated engagement, tapered-body compression, and the degree of implant–osteotomy interference may have a greater effect on rotational resistance during insertion. Previous studies have similarly reported that tapered implant bodies, progressive thread profiles, condensing designs, drilling undersizing, and bone density can affect insertion torque and primary mechanical engagement [6,7,8,9,11,12,13,14,15,16,17,18,22,23]. The present findings are consistent with this general biomechanical concept, but they should be interpreted within the specific artificial bone models and drilling protocols used in this study.

In the weak bone model, the KS 3 implant demonstrated higher maximum insertion torque and higher final insertion torque than the TSIII implant (Table 1, Figure 3). The increase from 18.61 to 22.51 Ncm in maximum insertion torque and from 13.59 to 16.82 Ncm in final insertion torque suggests that the KS 3 implant showed greater resistance during advancement in the low-density substrate under the tested drilling condition. Because weak bone provides reduced structural restraint during implant insertion, the observed increase may indicate greater implant–substrate contact and compression within the prepared osteotomy under the tested condition. Thus, the weak bone findings suggest that implant geometry-related differences may influence insertion resistance in low-support artificial bone conditions. This interpretation is consistent with previous studies reporting that tapered implant bodies, thread configuration, and condensing macrogeometries can increase insertion torque in low-density substrates [6,7,8,11,12,13,14,15,16,17,18].

In the standardized extraction-socket-like reduced-support model, the KS 3 implant also demonstrated higher torque values at the tested insertion depth of 5 mm (Table 2, Figure 4). In Case 1, a marked increase was observed, with both maximum and final insertion torque rising from 7.9 to 17.2 Ncm. In Case 2, both values increased from 17.6 to 21.3 Ncm. These results suggest that the KS 3 implant design produced higher insertion resistance under the tested standardized reduced-support geometry. However, the findings from the standardized extraction-socket-like reduced-support model should be interpreted with some caution. Previous studies on immediate implant placement have also indicated that socket morphology, apical engagement, and residual bone support can influence primary mechanical stability, which supports cautious interpretation of simplified in vitro socket-like models [19,20]. Therefore, the present data support a difference in insertion torque behavior under the tested 5 mm insertion-depth condition, rather than direct evidence of improved performance in all immediate-placement situations. Accordingly, the extraction-socket-like model should not be interpreted as reproducing a clinical extraction socket, but as a standardized reduced-support configuration for comparing torque–depth behavior under controlled conditions.

In the maxillary sinus model, the KS 3 implant showed higher final insertion torque than the TSIII implant at all residual bone heights under both CAS drilling and bone compaction drilling (Table 3 and Table 4; Figure 5 and Figure 6). These findings may be mechanically relevant within the tested sinus simulation model because residual bone height has been reported to influence implant stability in sinus-related placement models, and limited residual bone support can modify insertion resistance [15,17]. However, the present sinus simulation model cannot dissociate the isolated effect of implant macrodesign from its interaction with the fixed undersized osteotomy protocol. In the CAS drilling condition, a Ø2.8 mm final osteotomy was used for Ø4.0 mm implants, producing substantial implant–osteotomy interference. In the bone compaction condition, an F4.0 × 5 compaction drill was used according to the manufacturer’s protocol. Therefore, the higher torque observed for the KS 3 implant should be interpreted as insertion torque behavior under the tested fixed undersized drilling protocols, not as proof that macrodesign alone caused the observed difference. When available bone height is limited, implant geometry, residual bone height, artificial bone substrate, and drilling protocol may jointly influence the torque profile observed during implant placement.

The higher final insertion torque observed in the maxillary sinus model may be related to differences in external fixture geometry between the TSIII and KS 3 implants under the tested undersized drilling conditions. In the present comparison, the KS 3 implant had a greater core taper and greater lower thread depth than the TSIII implant, while both implants had the same nominal dimension, maximum outer diameter, upper thread depth, and SA surface treatment. Previous studies have reported that tapered implant bodies, thread configuration, and condensing macrogeometry can influence insertion torque, particularly in low-density or reduced-support substrates [6,7,8,11,12,13,14,15,16,17,18]. Therefore, the observed torque pattern is biomechanically plausible. However, because no finite element analysis, strain mapping, or direct bone-deformation measurement was performed, this explanation should be regarded only as a mechanistic interpretation consistent with the observed torque pattern, not as direct evidence of the underlying deformation mechanism.

The compaction-drill findings are also noteworthy. In the present study, final insertion torque values under bone compaction drilling were numerically higher than those under CAS drilling across the tested conditions, and higher final insertion torque values for the KS 3 implant were observed under both drilling protocols (Figure 5 and Figure 6). This finding suggests that implant geometry and the osteotomy protocol may have interacted to influence final insertion torque under the tested conditions. In other words, the between-implant torque difference was observed under both drilling approaches, but was maintained even when the surrounding bone was mechanically condensed prior to insertion. This consistency supports the interpretation that the observed torque response reflected an interaction among implant geometry, final osteotomy diameter, residual bone height, and drilling protocol under the tested conditions. Previous studies have also shown that osteotomy undersizing, drilling sequence, and bone compaction protocols can modify torque generation and bone deformation at the implant–bone interface [9,16,22,23].

The torque–depth integral analysis, which has been proposed as a full-curve parameter reflecting cumulative insertion resistance, revealed an important dissociation between cumulative insertion resistance and final insertion torque [9]. In Case 2 of the standardized extraction-socket-like reduced-support model, the TSIII implant showed a higher torque–depth integral, whereas the KS 3 implant showed a higher final insertion torque. This finding suggests that the two implants generated qualitatively different torque–depth profiles rather than simply different magnitudes of insertion torque. Biomechanically, the torque–depth integral may better approximate cumulative mechanical work against the substrate than peak or final torque alone, whereas final insertion torque reflects resistance at the final seating position [9]. Thus, a higher torque–depth integral may reflect greater cumulative resistance along the insertion path, whereas a higher final insertion torque may reflect a steeper increase in resistance during the late phase of seating. Therefore, higher final insertion torque should not be interpreted as uniformly better biomechanical performance. A higher final torque combined with a lower cumulative integral may indicate more localized resistance near the final seating region, which may be relevant when considering substrate deformation, heat generation, or excessive compression in living bone. Because the present study used artificial bone models and did not measure bone strain, temperature change, or biological tissue response, this interpretation remains mechanistic and should be validated in future studies.

Accordingly, the torque–depth integral was treated as an exploratory full-curve parameter rather than as a replacement for the predefined maximum and final insertion torque outcomes.

Several limitations should be acknowledged. First, the study was performed in artificial bone models and therefore could not fully reproduce the biologic, thermal, and anatomic complexity of the clinical environment. An a priori sample size calculation was not performed, and the small number of implants per condition limits the statistical power and generalizability of the findings. Therefore, the statistical results should be interpreted as exploratory and hypothesis-generating rather than confirmatory. In addition, although the polyurethane foam blocks were purchased for the same experimental series using the catalogue numbers listed in the Supplementary Table S3, the lot identifiers were not retained in the experimental records; therefore, lot-level material variability could not be independently verified. Very low within-condition variability was observed in some maxillary sinus model cells. Although the replicate-level raw torque–depth recordings are provided as a machine-readable Supplementary File and the measurement setup was mechanically guided and highly standardized, these low standard deviations should be interpreted cautiously. They may reflect the constrained nature of the automated laboratory setup, the fixed insertion axis, uniform drilling protocol, and controlled acquisition conditions, but they may also limit the generalizability of the inferential statistics. Therefore, the ANOVA and pairwise statistical outputs for the sinus model should be regarded as setup-specific exploratory descriptors rather than as definitive estimates of population-level effects. Second, different model categories involved different drilling protocols and comparator structures, which should be considered when interpreting consistency across models. In particular, the maxillary sinus simulation model used fixed undersized osteotomy protocols, and therefore the observed differences cannot be attributed to isolated macrodesign effects alone. The sinus findings should instead be interpreted as the combined response of implant geometry, residual bone height, artificial bone substrate, and the selected drilling protocol. Third, the present study did not evaluate bone strain, microdamage, heat generation, or pressure-related biologic responses associated with excessive compression. Therefore, higher insertion torque should not be interpreted as uniformly beneficial, because excessive compression may have adverse biologic effects in clinical bone. Fourth, because all insertions were performed by a single trained operator using a mechanically guided insertion setup, inter-operator reliability could not be assessed. This should be considered when interpreting reproducibility in broader clinical or laboratory settings. Finally, insertion torque was used here as a biomechanically relevant parameter, but it should not be regarded as the sole determinant of primary stability or long-term clinical success, because the relationship between insertion torque, micromotion, osseointegration, and clinical outcome can be influenced by bone quality, implant geometry, and measurement method [3,4,5,6,7,8,9,10]. Although the torque–depth integral was additionally calculated, it was treated as an exploratory secondary parameter and should be validated in future studies together with resonance frequency analysis, micromotion measurement, or removal torque testing.

Future studies should include validation in larger datasets, correlation with resonance frequency analysis or micromotion-related indices where possible, and additional fatigue or preclinical evaluation under more biologically relevant conditions. In particular, future sinus-model experiments should include osteotomy conditions that conform more closely to conventional Ø4.0 implant drilling protocols to help separate isolated implant-geometry effects from drilling-protocol interactions. Clinical confirmation will also be required to determine whether the observed insertion torque differences have clinical relevance in weak bone, immediate-placement, or transcrestal sinus-related applications.

Overall, the present results indicate that the two implant systems generated different insertion torque responses under the tested reduced-support artificial bone conditions, with higher maximum and/or final insertion torque values observed for the KS 3 implant in the tested models. Within the limitations of the study, these findings should be interpreted as exploratory mechanical data reflecting the interaction among implant geometry, artificial bone support, and osteotomy preparation, particularly in the maxillary sinus simulation model. These differences should be interpreted as exploratory mechanical findings reflecting the interaction among implant geometry, artificial bone support, and osteotomy preparation, particularly in the maxillary sinus simulation model.

## 5. Conclusions

Within the limitations of this controlled in vitro study, the KS 3 implant showed higher insertion torque values than the TSIII implant in standardized artificial bone models representing weak bone, a standardized extraction-socket-like reduced-support condition, and maxillary sinus simulation conditions. Torque–depth integral analysis provided additional exploratory information, indicating that cumulative insertion resistance and final seating torque may represent different aspects of insertion behavior. Therefore, the present findings should be interpreted as in vitro insertion torque data under the tested artificial bone and fixed drilling conditions, not as evidence of clinical superiority or as proof of an isolated macrodesign effect. In particular, the sinus model findings reflect the combined influence of implant geometry, residual bone height, and the selected undersized drilling protocol.

## Figures and Tables

**Table 1:** Insertion torque values in the weak bone model (n = 5).

Bone Condition	Implant	Maximum Insertion Torque (Ncm)	Final Insertion Torque (Ncm)
Soft bone (cortical #20, 1.0 mm + cancellous #10)	TSIII Ø4.0 SA Implant	18.61 ± 0.36	13.59 ± 0.22
Soft bone (cortical #20, 1.0 mm + cancellous #10)	KS 3 Ø4.0 SA Implant	22.51 ± 0.93	16.82 ± 0.39
Mean difference	KS3–TSIII	3.90	3.23
95% CI		2.77–5.03	2.74–3.72
*p*-value	Welch’s *t*-test	0.00026	0.0000025

**Table 2:** Insertion torque values in the standardized extraction-socket-like reduced-support model at a 5 mm insertion depth (n = 4).

Case	Bone Condition	Implant	Maximum Insertion Torque (Ncm)	Final Insertion Torque (Ncm)
Case 1	cortical #20, 1.0 mm + cancellous #10	TSIII Ø4.0 SA Implant	7.89 ± 0.68	7.89 ± 0.68
Case 1	cortical #20, 1.0 mm + cancellous #10	KS 3 Ø4.0 SA Implant	17.13 ± 0.44	17.13 ± 0.44
Case 2	cancellous #20	TSIII Ø4.0 SA Implant	17.60 ± 0.46	17.60 ± 0.46
Case 2	cancellous #20	KS 3 Ø4.0 SA Implant	21.32 ± 0.26	21.32 ± 0.26

**Table 3:** Maximum and final insertion torque values in the maxillary sinus model with CAS drilling (n = 4).

Residual Bone Height (mm)	Outcome	TSIII Implant (Ncm)	KS 3 Implant (Ncm)	Mean Difference	95% CI of Difference
2	Maximum	14.64 ± 0.53	17.87 ± 0.30	3.23	2.43–4.03
	Final	14.04 ± 0.39	17.26 ± 0.36	3.22	2.57–3.87
3	Maximum	17.31 ± 0.46	23.21 ± 0.01	5.90	5.17–6.63
	Final	17.29 ± 0.44	23.20 ± 0.00	5.91	5.21–6.61
4	Maximum	18.26 ± 0.03	22.66 ± 0.56	4.40	3.51–5.29
	Final	18.26 ± 0.03	22.66 ± 0.56	4.40	3.51–5.29
5	Maximum	18.88 ± 0.19	24.19 ± 0.52	5.31	4.52–6.10
	Final	18.88 ± 0.19	24.19 ± 0.52	5.31	4.52–6.10

Values are presented as mean ± standard deviation. Pairwise *p*-values are not reported because the between-implant comparisons at each residual bone height were treated as descriptive rather than confirmatory analyses. The primary model-level analysis for the sinus model was based on two-way ANOVA and was interpreted cautiously because of the limited sample size and low within-condition variability observed in some cells. Additional statistical outputs are provided in the Supplementary Materials. Very small standard deviations in terminal final-torque values should be interpreted as numerical dispersion of exported endpoint readings rather than metrological precision beyond the specified accuracy of the torque sensor.

**Table 4:** Maximum and final insertion torque values in the maxillary sinus model with bone compaction drilling (n = 4).

Residual Bone Height (mm)	Outcome	TSIII Implant (Ncm)	KS 3 Implant (Ncm)	Mean Difference	95% CI of Difference
2	Maximum	14.51 ± 0.26	18.87 ± 0.31	4.36	3.86–4.86
	Final	14.51 ± 0.26	18.76 ± 0.35	4.25	3.71–4.79
3	Maximum	18.41 ± 0.86	23.81 ± 0.09	5.40	4.04–6.76
	Final	18.41 ± 0.86	23.77 ± 0.01	5.35	3.99–6.73
4	Maximum	18.70 ± 0.06	25.42 ± 0.50	6.72	5.93–7.51
	Final	18.70 ± 0.06	25.41 ± 0.51	6.71	5.91–7.51
5	Maximum	19.52 ± 0.09	25.46 ± 0.38	5.94	5.35–6.53
	Final	19.52 ± 0.09	24.68 ± 0.08	5.16	5.01–5.31

Values are presented as mean ± standard deviation. Pairwise *p*-values are not reported because the between-implant comparisons at each residual bone height were treated as descriptive rather than confirmatory analyses. The primary model-level analysis for the sinus model was based on two-way ANOVA and was interpreted cautiously because of the limited sample size and low within-condition variability observed in some cells. Additional statistical outputs are provided in the Supplementary Materials. Very small standard deviations in terminal final-torque values should be interpreted as numerical dispersion of exported endpoint readings rather than metrological precision beyond the specified accuracy of the torque sensor.

## Data Availability

The replicate-level raw torque–depth data supporting the findings of this study are provided as a machine-readable Supplementary File in CSV format. The file includes the unprocessed depth–torque recordings for each independent implant insertion and allows verification of the maximum insertion torque, final insertion torque, torque–depth integral, mean, and standard deviation values reported in the manuscript and Supplementary Table S1. The dataset will also be made available through a persistent public repository upon publication if required by the journal.

## Supplementary Materials

The following supporting information can be downloaded at: https://www.mdpi.com/article/10.3390/bioengineering13060705/s1.
