# Supplementary material for: Insertion Torque Characteristics of the KS 3 Implant in Weak Bone, Standardized Extraction-Socket-like, and Maxillary Sinus Simulation Models: An In Vitro Comparative Study
Source: Bioengineering (Basel). 2026 Jun 19;13(6):705. doi: 10.3390/bioengineering13060705 (PMC13295728; doi:10.3390/bioengineering13060705)
Supplement: Supplementary file 1 [file bioengineering-13-00705-s001.zip › Supplementary Tables.pdf]

Supplementary Table S1. Torque–depth integral values calculated from recorded torque–depth curves.

Model	Condition	Drilling protocol	Insertion/ residual condition	N (TSIII)	TSIII torque–depth integral (Ncm·mm)	N (KS 3)	KS 3 torque–depth integral (Ncm·mm)	Mean difference (KS 3 − TSIII)	95% CI of difference	Welch's p-value	Individual TSIII values	Individual KS 3 values
**Weak bone**	Soft bone (#20 cortical 1.0 mm + #10 cancellous)	—	—	5	73.65 ± 2.33	5	85.39 ± 3.43	11.74	7.36 to 16.12	3.84E-4	76.91, 71.03, 72.39, 72.88, 75.06	81.45, 86.76, 90.53, 84.22, 84.01
**Extraction-socket-like reduced-support**	Case 1 (cortical #20 1.0 mm + cancellous #10)	—	5 mm insertion depth	4	23.67 ± 1.70	4	34.66 ± 0.49	10.99	8.40 to 13.59	5.12E-4	21.65, 25.80, 23.53, 23.69	34.80, 34.17, 35.27, 34.40
	Case 2 (cancellous #20 only)	—	5 mm insertion depth	4	40.42 ± 1.63	4	34.34 ± 0.24	-6.07	-8.64 to -3.50	4.50E-3	38.12, 41.09, 41.92, 40.53	34.63, 34.05, 34.29, 34.40
**Maxillary sinus simulation**	Residual bone height 2 mm	CAS drilling	—	4	76.57 ± 2.61	4	95.90 ± 0.80	19.33	15.35 to 23.32	2.96E-4	73.61, 79.97, 76.15, 76.54	94.99, 96.94, 95.77, 95.89
	Residual bone height 3 mm	CAS drilling	—	4	84.80 ± 0.95	4	102.15 ± 0.05	17.35	15.84 to 18.87	4.40E-5	83.72, 86.04, 84.65, 84.79	102.21, 102.08, 102.16, 102.15
	Residual bone height 4 mm	CAS drilling	—	4	78.52 ± 0.28	4	89.08 ± 2.45	10.56	6.69 to 14.42	3.00E-3	78.21, 78.89, 78.48, 78.52	86.31, 92.26, 88.69, 89.05
	Residual bone height 5 mm	CAS drilling	—	4	73.72 ± 0.48	4	91.30 ± 1.93	17.58	14.61 to 20.56	1.93E-4	74.26, 73.10, 73.80, 73.73	89.12, 93.81, 91.00, 91.28
	Residual bone height 2 mm	Bone compaction drilling	—	4	68.49 ± 0.22	4	102.83 ± 0.40	34.34	33.73 to 34.95	9.42E-10	68.73, 68.21, 68.58, 68.42	102.38, 103.32, 102.66, 102.94
	Residual bone height 3 mm	Bone compaction drilling	—	4	89.31 ± 5.29	4	120.06 ± 1.97	30.75	22.76 to 38.75	5.20E-4	94.82, 88.88, 82.26, 91.26	117.88, 122.48, 119.26, 120.64
	Residual bone height 4 mm	Bone compaction drilling	—	4	88.01 ± 1.12	4	111.81 ± 1.81	23.79	21.06 to 26.53	3.00E-6	89.25, 86.64, 88.47, 87.69	109.79, 114.03, 111.06, 112.33
	Residual bone height 5 mm	Bone compaction drilling	—	4	80.45 ± 0.32	4	98.34 ± 1.74	17.89	15.16 to 20.61	1.74E-4	80.81, 80.05, 80.58, 80.36	100.28, 96.19, 99.05, 97.83

Values are presented as mean ± standard deviation. Torque–depth integrals were calculated from the recorded torque–depth curves using the trapezoidal rule after adding a zero-torque value at 0 mm. Individual values represent replicate-level torque–depth integrals. Welch's p-values were used for between-implant comparisons within each experimental condition.

Supplementary Table S2. Materials, instruments, and product information used in the study.

Item	Product	Manufacturer	City/Country	Catalogue No.
**Implant**	TSIII SA Implant	Osstem Implant Co., Ltd.	Seoul, Republic of Korea	TS3S4010S
**Implant**	KS 3 SA Implant	Osstem Implant Co., Ltd.	Seoul, Republic of Korea	KS3S4010S
**Artificial bone**	Sawbones polyurethane foam block	Pacific Research Laboratories	Vashon, WA, USA	1522-105, 1522-751, 1522-1199
**Side-cut drill**	Ø2.0/2.5 × 10 Side-cut drill	Osstem Implant Co., Ltd.	Seoul, Republic of Korea	OSLMD20L
**Taper drill**	F3.5 × 10 122 Taper 3510 drill	Osstem Implant Co., Ltd.	Seoul, Republic of Korea	122TPD3510
**CAS drill**	Ø2.8 CAS drill	Osstem Implant Co., Ltd.	Seoul, Republic of Korea	OSNDR2813TL
**Compaction drill**	F4.0 × 5 compaction drill	Osstem Implant Co., Ltd.	Seoul, Republic of Korea	BCD4005
**Torque sensor**	Torque sensor	Kistler	Winterthur, Switzerland	4502A

Supplementary Table S3. Information of sawbones polyurethane foam block.

	Cortical bone thickness	Cancellous bone thickness	Hounsfield units	Catalogue No.
**Hard**	3.0 mm (#50)	> 25 mm (#30)	1,250	1522-105
**Normal**	1.5 mm (#40)	> 25 mm (#20)	850 ~ 1,250	1522-751
**Soft**	1.0 mm (#20)	> 25 mm (#10)	350 ~ 850	1522-1199

Supplementary Table S4. Density of sawbones polyurethane foam block.

	Density	
	**lb/ft^3**	**g/cc**
**#10**	10	0.16
**#20**	20	0.32
**#30**	30	0.48
**#40**	40	0.64
**#50**	50	0.80
